# Piperlongumine Induces Apoptosis and Synergizes with Cisplatin or Paclitaxel in Human Ovarian Cancer Cells

**DOI:** 10.1155/2014/906804

**Published:** 2014-05-08

**Authors:** Li-Hua Gong, Xiu-Xiu Chen, Huan Wang, Qi-Wei Jiang, Shi-Shi Pan, Jian-Ge Qiu, Xiao-Long Mei, You-Qiu Xue, Wu-Ming Qin, Fei-Yun Zheng, Zhi Shi, Xiao-Jian Yan

**Affiliations:** ^1^Department of Gynecology, The First Affiliated Hospital of Wenzhou Medical College, Shangcai Village South, Ouhai District, Wenzhou, Zhejiang 325000, China; ^2^Department of Gynecology, The Third Affiliated Hospital of Sun-Yat Sen University, Guangzhou, Guangdong 510632, China; ^3^Department of Cell Biology and Institute of Biomedicine, College of Life Science and Technology, Jinan University, Room 708, The 2nd Engineer and Scientific Building, 601 Huangpu RoadWest, Guangzhou, Guangdong 510632, China

## Abstract

Piperlongumine (PL), a natural alkaloid from *Piper longum L.*, possesses the highly selective and effective anticancer property. However, the effect of PL on ovarian cancer cells is still unknown. In this study, we firstly demonstrate that PL selectively inhibited cell growth of human ovarian cancer cells. Furthermore, PL notably induced cell apoptosis, G2/M phase arrest, and accumulation of the intracellular reactive oxidative species (ROS) in a dose- and time-dependent manner. Pretreatment with antioxidant N-acety-L-cysteine could totally reverse the PL-induced ROS accumulation and cell apoptosis. In addition, low dose of PL/cisplatin or paclitaxel combination therapies had a synergistic antigrowth effect on human ovarian cancer cells. Collectively, our study provides new therapeutic potential of PL on human ovarian cancer.

## 1. Introduction

Ovarian cancer is the most lethal cancer of female reproductive tract, accounting for ~16,000 deaths annually [1]. The high mortality results partially from the nonspecific and commonly misinterpreted symptoms associated with the disease. As a result, more than 70% of patients are diagnosed only after the disease has progressed to a late stage [2]. Cytoreduction surgery combined with cisplatin (DDP) or paclitaxel (TX) chemotherapy in ovarian cancer results in a clinical remission but is infrequently a cure. Improving the current responses to chemotherapy is a key for achieving a better outcome and we have demonstrated that silence of survivin could effectively increase the sensitivity of ovarian cancer cells to chemotherapeutical drugs [3–6]. Etiology of ovarian cancer is still unknown; several theories such as gonadotropin theory and genetic alteration have been proposed as the mechanism of carcinogenesis [7]. A role for chronic oxidative stress has been proposed in the etiology of malignant transformation and elevation of reactive oxygen species (ROS) levels has been observed in many cancer cells relative to nontransformed cells [8, 9]. Therefore, the elevated ROS in cancer cells provide for a prospect of selective cancer treatment [10, 11].

**Figure 1:**
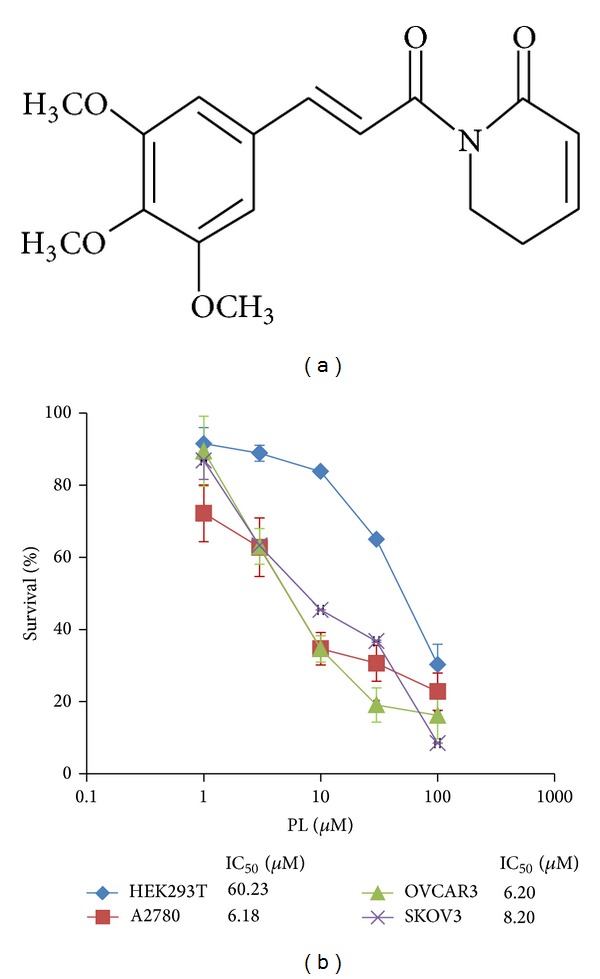
PL selectively inhibited the growth of human ovarian cancer cells. (a) Structure of PL. (b) The growth curves of PL-treated A2780, OVCAR3, SKOV3, and HEK293T cells. Cells were grown in 96-well plates for 24 hr and treated with PL (0, 1, 3, 10, 30, and 100 *μ*M) for 72 hr. Cell survival was measured by MTT assay and the IC_50_ value of PL in each cell lines was calculated.

Piperlongumine (PL) is a biologically active alkaloid isolated from the long pepper (*Piper longum* Linn) which is used to treat cough, respiratory infections, stomachache, and other diseases in Indian Ayurvedic medicine [12]. The chemical structure of PL has been well-characterized (Figure 1(a)). Recently, PL has shown potential cytotoxic and antitumor properties on several types of cancer cells, including hematological [13], gastrointestinal [14], central nervous system [15], and other solid tumors [16]. Its cytotoxicity was observed in the micromolar range in tumor cells, but not in normal cells [14, 16–18]. Quantitative proteomics approaches identified two strong PL-binding proteins, S-transferase pi 1 (GSTP1) and carbonyl reductase 1, known to regulate oxidative stress by modulating redox and ROS homeostasis [18]. Consistent with this theory, when PL interacted directly with GSTP1, protein glutathionylation was identified as a process associated with cellular toxicity [19]. Furthermore, PL induced cell cycle arrest in G1 or G2/M phase followed by mitochondrial-dependent apoptosis [20]. More recently, PL also promoted autophagy and mediates cell death in several cancer cell lines [21, 22].

In the present study, we firstly demonstrate that PL selectively inhibited cell growth and induced ROS-dependent cell apoptosis and G2/M cell cycle arrest in human ovarian cancer. Furthermore, PL synergizes with DDP and TX to inhibit the growth of human ovarian cancer cells. Our results provide new drug therapeutic potential of PL on human ovarian cancer.

## 2. Materials and Methods

### 2.1. Cells Lines and Reagents

The human epithelia ovarian cancer (EOC) lines A2780, OVCAR3, and SKOV3 and human embryonic kidney cell line HEK293T were cultured in DMEM (Gibco, NY, USA) culture medium supplemented with 10% fetal bovine serum (Gibco, NY, USA) at 37°C and 5% CO_2_. PL, N-acetyl-L-cysteine (NAC), dihydroethidium (DHE), anti-*β*-actin antibody, and other chemicals were purchased from Sigma Chemical Co. (St. Louis, MO, USA). Anti-cleaved-PARP (C-PARP) antibody was from Cell Signaling Technologies (Danvers, MA, USA).

### 2.2. MTT Assay

Cells were harvested with trypsin and resuspended in a final concentration of 5 × 10^4^ cells/mL. Aliquot (100 *μ*L) for each cell suspension was distributed evenly into 96-well multiplates. The different concentrations of PL (10 *μ*L/well) were added to designated wells. After 72 hours (hr), 10 *μ*L of 3-(4,5-dimethylthiazol-2yl)-2,5-diphenyl tetrazolium bromide (MTT) solution (5 mg/mL) was added to each well, and the plate was further incubated for 4 hr, allowing viable cells to change the yellow MTT into dark-blue formazan crystals. Subsequently, the medium was discarded and 100 *μ*L of dimethylsulfoxide (DMSO) was added to each well to dissolve the formazan crystals. The absorbance in individual well was determined at 490 nm by multidetection microplate reader 680 (BioRad, PA, USA). The concentrations required to inhibit growth by 50% (IC_50_) were calculated from survival curves using the Bliss method [23, 24]. For drug combination experiments, cells were cotreated with different concentrations of PL and DDP or TX for 72 hr. The data were analyzed by CompuSyn software with the results showed in combination index (CI) values, where CI <1, =1, and >1 indicate synergism, additive effect, and antagonism, respectively.

### 2.3. Apoptosis Analysis

Cell apoptosis was evaluated with flow cytometry (FCM) assay. Briefly, cells were harvested and washed twice with cold phosphate-buffered saline (PBS), stained with Annexin V-FITC and propidium iodide (PI) in the binding buffer, and detected by FACSCalibur FCM (BD, CA, USA) after 15 min incubation at room temperature in the dark. Fluorescence was measured at an excitation wave length of 480 nm through FL-1 (530 nm) and FL-2 (585 nm) filters. The early apoptotic cells (Annexin V positive only) and late apoptotic cells (Annexin V and PI positive) were quantified.

### 2.4. Measurement of ROS Production

Cells were incubated with 10 *μ*M of DHE for 30 min at 37°C, washed twice with PBS, and immediately microphotographed under a conventional fluorescent microscope (Olympus, Japan). For each well, 5 fields were taken randomly.

### 2.5. Cell Cycle Analysis

Cells were harvested and washed twice with cold PBS and then fixed with ice-cold 70% (v/v) ethanol for 30 min at 4°C. After centrifugation at 200 ×g for 10 min, cells were washed twice with PBS, resuspended with 0.5 mL PBS containing PI (50 *μ*g/mL), Triton X-100 (0.1%, v/v), 0.1% sodium citrate, and DNase-free RNase (100 *μ*g/mL), and detected by FCM after 15 min incubation at room temperature in the dark. Fluorescence was measured at an excitation wave length of 480 nm through a FL-2filter (585 nm). Data were analyzed using ModFit LT 3.0 software (Becton Dickinson).

### 2.6. Western Blot Analysis

Cells were harvested and washed twice with cold PBS and then resuspended and lysed in RIPA buffer (1% NP-40, 0.5% sodium deoxycholate, 0.1% SDS, 10 ng/mL PMSF, 0.03% aprotinin, and 1 *μ*M sodium orthovanadate) at 4°C for 30 min. Lysates were centrifuged for 10 min at 14,000 ×g and supernatants were stored at −80°C as whole cell extracts. Total protein concentrations were determined with Bradford assay. Proteins were separated on 12% SDS-PAGE gels and transferred to polyvinylidene difluoride membranes. Membranes were blocked with 5% BSA and incubated with the indicated primary antibodies. Corresponding horseradish peroxidase-conjugated secondary antibodies were used against each primary antibody. Proteins were detected using the chemiluminescent detection reagents and films.

### 2.7. Statistical Analysis

All experiments were repeated at least 3 times and the differences were determined by using Student's *t*-test. The significance was determined at *P* < 0.05.

## 3. Results

### 3.1. PL Selectively Inhibited the Growth of Ovarian Cancer Cells

To determine the effect of PL on ovarian cancer cells, three ovarian cancer cell lines A2780, OVCAR3, and SKOV3 and human embryonic kidney cell line HEK293T were treated with either the vehicle control (DMSO) or increasing concentrations of PL range from 1 *μ*M to 100 *μ*M for 72 hr. As shown in Figure 1(b), the results of MTT assay revealed that the growth of three ovarian cancer cell lines was similarly inhibited by PL in a concentration-dependent manner. The IC_50_ values of PL after 72 hr exposure were 6.18 *μ*M, 6.20 *μ*M, and 8.20 *μ*M in A2780, OVCAR3, and SKOV3, respectively (Figure 1(b)). However, PL showed the much weaker inhibition effect on the human normal HEK293T cells than three ovarian cancer cell lines and the IC_50_ values of PL were 60.23 *μ*M to HEK293T. These data suggested that PL selectively inhibits the growth of human ovarian cancer cells compared with normal cells.

### 3.2. PL Induced Apoptosis in OVCAR3 Ovarian Cancer Cells

**Figure 2:**
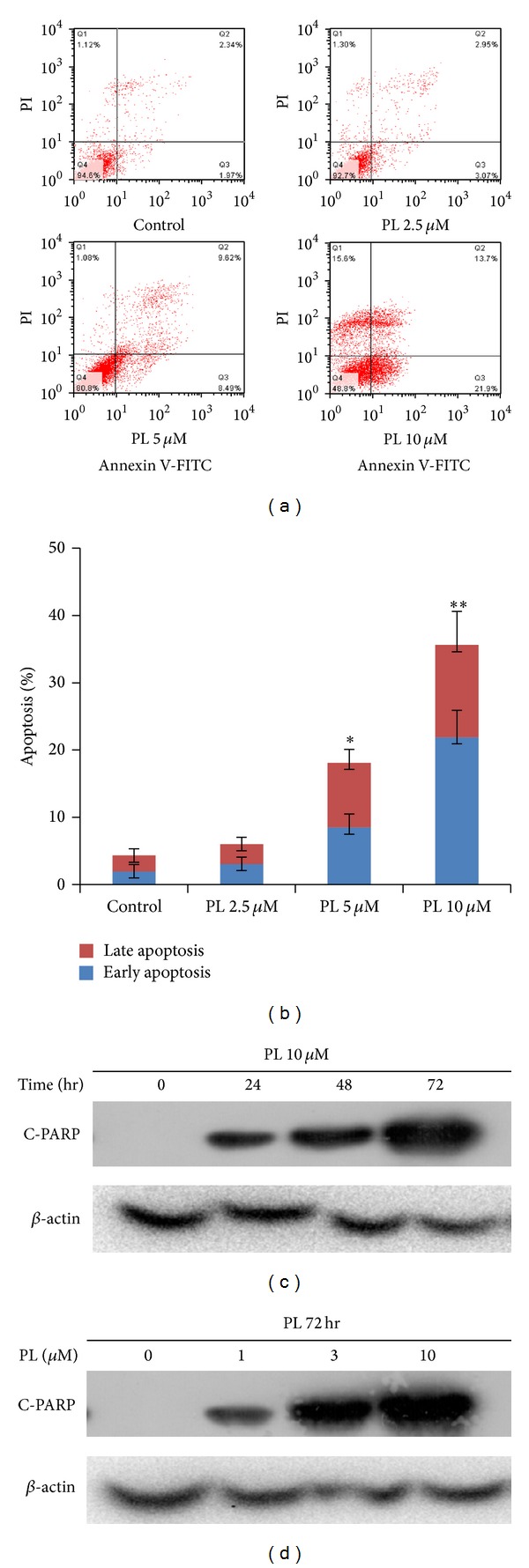
PL induced apoptosis in OVCAR3 ovarian cancer cells. (a) The results of cell apoptosis in PL-treated OVCAR3 cells. Cells were treated with the indicated concentration of PL for 48 hr, stained with Annexin V/PI, and examined by FCM. The proportions of Annexin V+/PI− and Annexin V+/PI+ cells indicated the early and late stages of apoptosis. (b) The quantified results of (a). (c) and (d) Representative Western blotting analysis of C-PARP in OVCAR3 cells treated with the indicated PL. *β*-actin was used as loading control. *P < 0.05 and **P < 0.01 versus corresponding control.

To determine whether the growth inhibition of ovarian cancer cells by PL was due to the induction of apoptosis, cell apoptosis was assessed by FCM with Annexin V/PI staining. OVCAR3 cells were treated with the different concentrations of PL for 48 hr, stained with Annexin V/PI, and examined by FCM. As shown in Figures 2(a) and 2(b), PL treatment mostly induced apoptosis in OVCAR3 cells and both proportions of Annexin V+/PI− (early stage of apoptosis) and Annexin V+/PI+ (late stage of apoptosis) cells were increased with the elevated PL concentrations.

To further detect the apoptosis induced by PL, the expression of apoptosis marker cleaved-PARP (C-PARP) proteins was analyzed by Western blot in OVCAR3 cells with or without PL treatment. Compared with the loading control *β*-actin proteins, the levels of C-PARP proteins in OVCAR3 cells were increased in a dose- and time-dependent manner after being treated with PL (Figures 2(c) and 2(d)). Together, these results indicated that the growth inhibition of PL on ovarian cancer cells might be due to the induction of apoptosis.

### 3.3. PL Induced SubG1 Accumulation and G2/M Arrest in OVCAR3 Ovarian Cancer Cells

**Figure 3:**
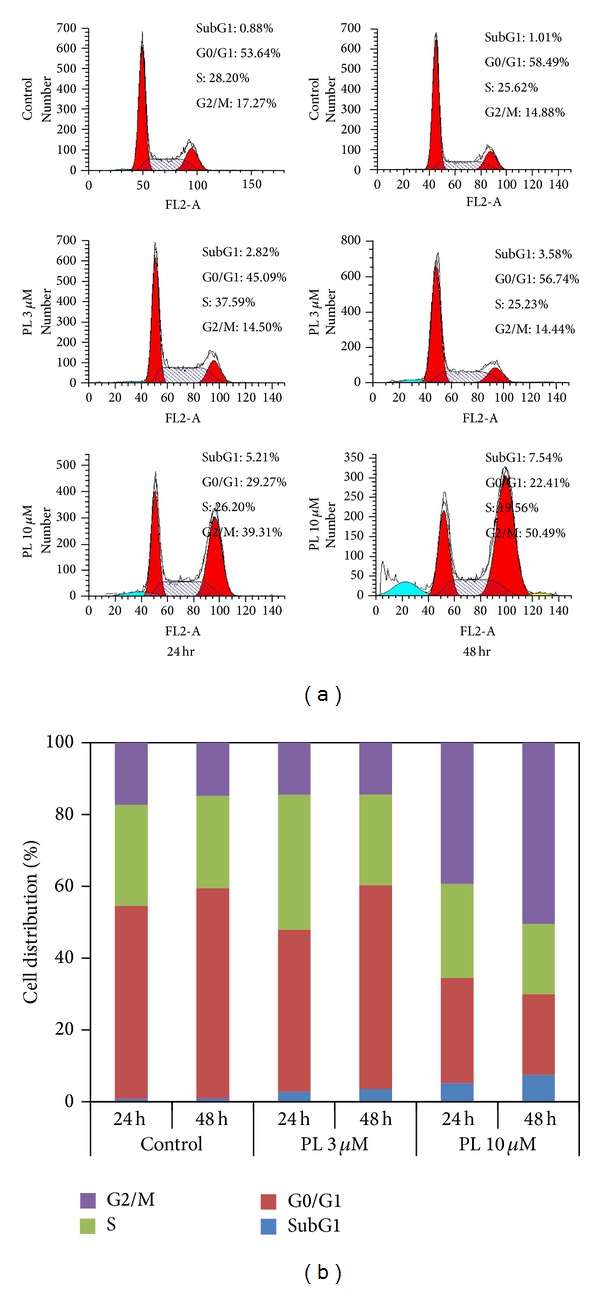
PL induced subG1 accumulation and G2/M arrest in OVCAR3 ovarian cancer cells. (a) The results of cell cycle distribution in PL-treated OVCAR3 cells. Cells were treated with the indicated PL, stained with PI, and examined by FCM. The percentages of subG1, G1/G0, S, and G2/M phase were calculated using ModFit LT 3.0 software. (b) The quantified results of (a).

In addition to the evaluation of PL-induced growth inhibition and proapoptosis effect, the effect of PL on cell cycle distribution was analyzed by FCM with PI staining. OVCAR3 cells were treated with PL (3 *μ*M and 10 *μ*M) for 24 hr and 48 hr, stained with PI, and examined by FCM. The percentages of subG1, G1/G0, S, and G2/M phase were calculated using ModFit LT 3.0 software. Compared to the control groups, the subG1 and G2/M groups of PL-treated OVCAR3 cells were dose- and time-dependently increased (Figures 3(a) and 3(b)). Therefore, the effect of PL on cell cycle distribution in OVCAR3 cells is the induction of subG1 accumulation which indicated apoptosis-associated chromatin degradation and arrest of cell cycle in G2/M phase.

### 3.4. ROS Generation Was Critical for PL-Induced Apoptosis in OVCAR3 Ovarian Cancer Cells

**Figure 4:**
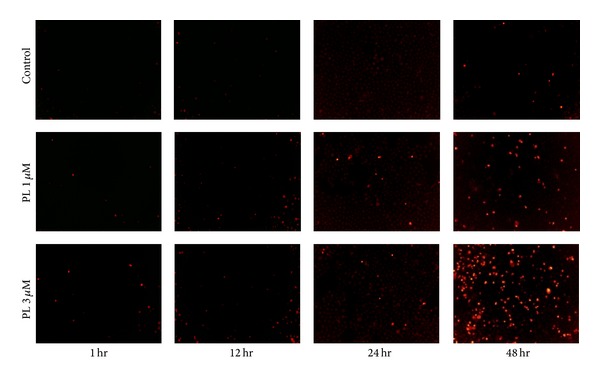
Piperlongumine induced ROS accumulation in OVCAR3 ovarian cancer cells. Cells were treated with PL as indicated, incubated with DHE, and microphotographed under a conventional fluorescent microscope.

**Figure 5:**
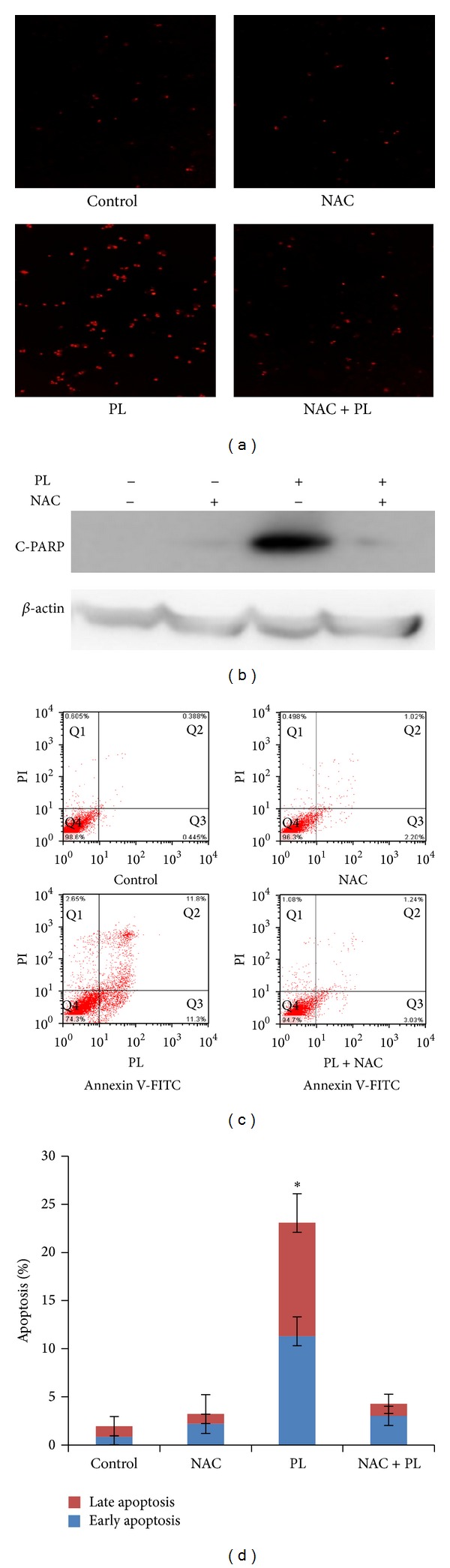
ROS generation was critical for PL-induced apoptosis in OVCAR3 ovarian cancer cells. (a) ROS accumulation and (c) cell apoptosis in PL-treated OVCAR3 cells were reversed by NAC. Cells were treated with PL (10 *μ*M) for 24 hr in the presence or absence of 3 mM NAC pretreatment for 1 hr. The apoptosis was detected by FCM with Annexin V/PI staining and the expression of C-PARP proteins was analyzed by Western blot. (c) The quantified results of (d). (b) Representative Western blotting analysis of C-PARP in OVCAR3 cells treated as indicated. *β*-actin was used as loading control. *P < 0.05 and **P < 0.01 versus corresponding control.

Numerous anticancer agents exhibit antitumor activity via ROS-dependent activation of apoptotic cell death [25] and it has previously been reported that the elevated intracellular ROS mediated PL-induced apoptosis in several human cancer cells (EJ, MDA-MB-231, U2OS, and MDA-MB-435) [18]. Dihydroethidium (DHE) is a classic ROS fluorescent probe, which can penetrate through living cell membrane freely and be oxidized by intracellular ROS to oxide ethidium that conjugated with DNA to emit the detectable red fluorescence. As shown in Figure 4, PL exposure resulted in a time- and concentration-dependent ROS accumulation in OVCAR3 cells. Significant intracellular ROS generation was observed when the cells were treated just for as little as 1 hr; ROS production was increasing and being maintained even at 48 hr, indicating a rapid and sustained generation of ROS in the PL-treated cells. As predicted, the PL-induced ROS accumulation was greatly reduced by NAC due to its ability to elevate intracellular glutathione to prevent the production of ROS (Figures 5(a) and 5(b)).

To further investigate the relationship between the ROS generation and PL-induced apoptosis, OVCAR3 cells were treated with PL (10 *μ*M) for 24 hr in the presence or absence of 3 mM NAC pretreatment for 1 hr. The apoptosis was detected by FCM with Annexin V/PI staining and the expression of C-PARP proteins was analyzed by Western blot. As shown in Figures 5(c) and 5(d), PL-induced apoptosis and the increased expression of C-PARP proteins were completely blocked by NAC. These data suggested that ROS generation is critical for PL-induced apoptosis in OVCAR3 ovarian cancer cells.

### 3.5. PL Synergized with DDP or TX in OVCAR3 Ovarian Cancer Cells

**Figure 6:**
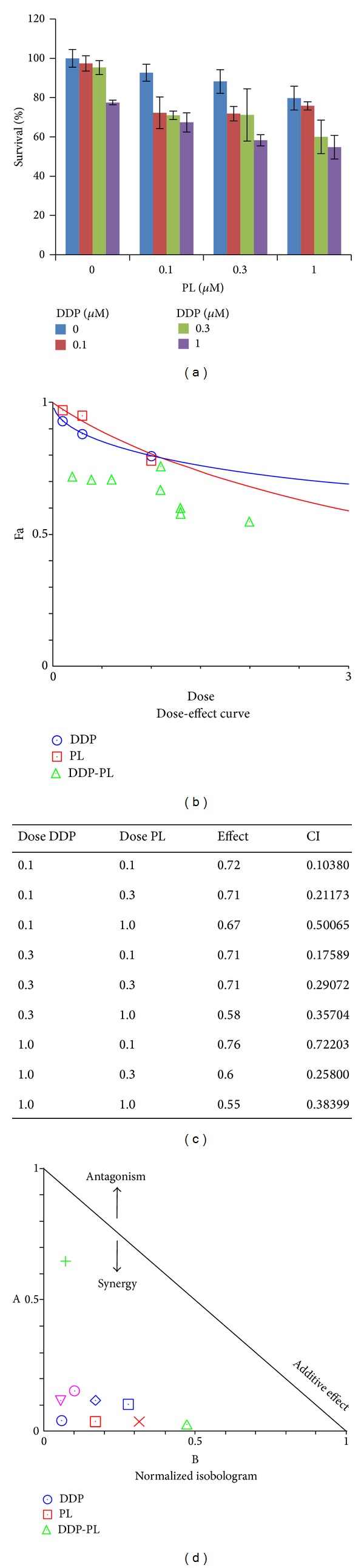
PL synergized with DDP in OVCAR3 ovarian cancer cells. (a) The growth histogram of OVCAR3 treated with the indicated PL and DDP. Cells were treated with PL (range from 0.1 to 1 *μ*M) combined with DDP (0.1 to 1 *μ*M) and cell survival was detected by MTT assay. The data were analyzed by CompuSyn software with the results showing dose-effect curve (b), CI values (c), and normalized isobologram (d).

**Figure 7:**
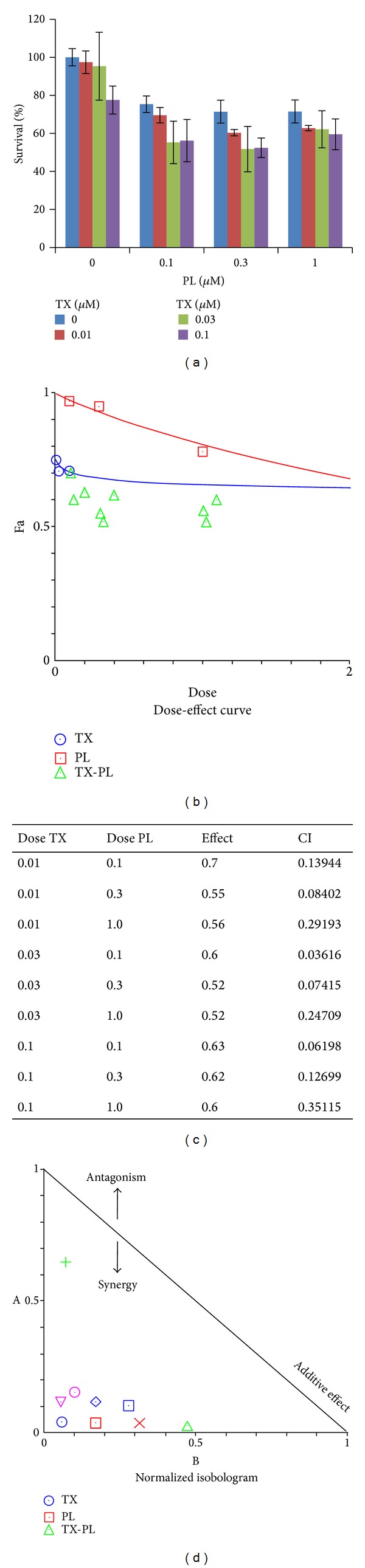
PL synergized with TX in OVCAR3 ovarian cancer cells. (a) The growth histogram of OVCAR3 treated with the indicated PL and TX. Cells were treated with PL (range from 0.1 to 1 *μ*M) combined with TX (0.01 to 0.1 *μ*M) and cell survival was detected by MTT assay. The data were analyzed by CompuSyn software with the results showing dose-effect curve (b), CI values (c), and normalized isobologram (d).

Combinations of agents at low doses can reduce side effects of chemotherapy and improve the compliance of patients with chemotherapy; thus investigating novel agents for combination chemotherapy to overcome drug resistance and achieve better therapeutic effects is of vital significance. For example, a new synthetic compound phenoxodiol exerted potent anticancer activity combined with DDP against ovarian cancer [26]. Currently, DDP and TX are the two of main chemotherapeutic drugs for ovarian cancer in clinic. The present study tested whether lower dose of two drugs in combination (PL + DDP or PL + TX) was able to exert a synergistic antitumor activity compared to PL, DDP, or TX treatment alone. OVCAR3 cells were treated with PL (range from 0.1 to 1 *μ*M) combined with DDP (0.1 to 1 *μ*M) or TX (0.01 to 0.1 *μ*M) and cell survival was detected by MTT assay. As shown in Figures 6 and 7, the cell survival was decreased in the combination of lower dose PL with either DDP or TX. The CI values of both combination were <1, suggesting that the antigrowth effect of combination is synergistic rather than additive. These observations demonstrated that PL was able to sensitize OVCAR3 ovarian cancer cells to both DDP and TX.

## 4. Discussion

In this report, we firstly demonstrated that PL selectively mediated time- and concentration-dependent antigrowth effects on human ovarian cancer cells. The IC_50_ value after 72 hr treatment with PL ranges from 6 to 8 *μ*M in three human ovarian cancer cell lines, was similar to the IC_50_ value of PL in other solid cancers [14]. The results of FCM analysis showed that PL treatment increased both early and later stage of apoptosis, subG1 accumulation and G2/M phase arrest. Inhibition of the intercellular ROS accumulation by NAC could totally block PL-induced apoptosis. Moreover, PL synergistically enhanced the antigrowth effect of DDP or TX, which suggested that PL might be a potential chemosensitizer for ovarian cancer chemotherapy.

The intracellular production of ROS greatly contributes to the regulation of cell survival and death [27]. Although cancer cells become well adapted to persistent intrinsic oxidative stress, a further increase in ROS above the toxic threshold level may result in cell death [28]. Chemotherapeutical agents including DDP, TX and etoposide induce apoptotic cell death by increasing the intracellular ROS levels. However, continuous DDP treatment may reduce cellular ROS levels and cancer cells may become drug resistant. The chemoresistance of ovarian cancer was also linked to increased cellular glutathione content [29]. Furthermore, an elevation of the cellular ROS level by exogenous ROS generation in combination with DDP resensitized drug-resistant cancer cells [30]. It has been postulated that PL kills carcinoma cells by targeting their “nononcogene codependency” on elevated antioxidative defense pathways acquired in response to cell transformation-induced oxidative stress [18]. Our findings on ovarian cancer cells have suggested that PL-mediated growth inhibition was related to G2/M phase arrest and apoptosis by the increasing intercellular ROS. Previously reported, a dose-dependent decrease of cdc-2 expression but not cyclinB1 changing was associated with PL-mediated cell cycle arrest in PC-3 cells [16]. The present study has attributed the generation of ROS to the proapoptotic effect of PL in ovarian cancer cells, which was in agreement with the previous findings in other cancer cell types [12, 18].

Altogether, the present study offers the first evidence that PL selectively inhibited cell growth and induced ROS-dependent cell apoptosis and G2/M cell cycle arrest in human ovarian cancer. Furthermore, PL synergizes with DDP and TX to inhibit the growth of human ovarian cancer cells. Further* in vivo* experiments may aid in the confirmation of the therapeutic efficacy of this agent for patients with ovarian cancer.
